# Deciphering microbial communities involved in marine steel corrosion using high‐throughput amplicon sequencing

**DOI:** 10.1111/1758-2229.70001

**Published:** 2024-08-27

**Authors:** Biji Shibulal, Martin Peter Smith, Ian Cooper, Heidi Marie Burgess, Norman Moles, Alison Willows

**Affiliations:** ^1^ School of Applied Sciences University of Brighton Brighton UK

## Abstract

To characterize the source and effects of bacterial communities on corrosion of intertidal structures, three different UK coastal sites were sampled for corrosion materials, sediment and seawater. Chemical analyses indicate the activity of sulfate‐reducing microbes (SRBs) at 2 sites (Shoreham and Newhaven), but not at the third (Southend‐on‐Sea). Microbial communities in the deep sediment and corrosion samples are similar. The phylum Proteobacteria is dominant (40.4% of the total ASV), followed by Campilobacterota (11.3%), Desulfobacterota and Firmicutes (4%–5%). At lower taxonomic levels, corrosion causing bacteria, such as *Shewanella* sp. (6%), *Colwellia* sp. (7%) and *Mariprofundus* sp. (1%), are present. At Southend‐on‐sea, the relative abundance of Campilobacterota is higher compared to the other two sites. The mechanism of action of microorganisms at Shoreham and Newhaven involves biogenic sulfuric acid corrosion of iron by the combined action of SRBs and sulfur‐oxidizing microbes. However, at Southend‐on‐sea, sulfur compounds are not implicated in corrosion, but SRBs and other electroactive microbes may play a role in which cathodic reactions (electrical MIC) and microbial enzymes (chemical MIC) are involved. To contribute to diagnosis of accelerated intertidal corrosion types, we developed a rapid identification method for SRBs using quantitative polymerase chain reaction high‐resolution melt curve analysis of the *dsr*B gene.

## Graphical abstract

Aggressive forms of intertidal steel corrosion are shown to involve complex microbial consortia including sulfate‐reducing, sulfide‐oxidizing and iron‐oxidizing bacteria along with others including electroactive microbes. Statistical analysis shows the populations are derived from local bed sediment, indicating sediment anoxia as a risk factor for accelerated microbially influenced corrosion.

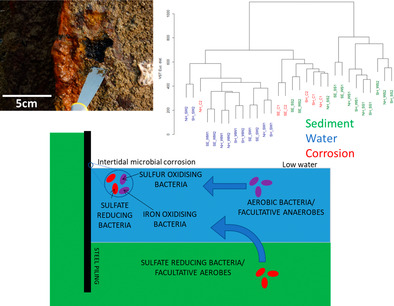

## INTRODUCTION

Biocorrosion or microbially influenced corrosion (MIC) is a complex process involving both abiotic factors (temperature, pH, salinity and other physical and chemical factors) and biotic factors (microbial metabolism). It is a global concern to many commercial sectors, and in particular offshore industry. MIC accounts for about 2.5 trillion dollars loss worldwide as estimated in 2015 (Ma et al., 2021). This occurs due to the synergistic action of the metabolism of different microbial species over the metal surface. Though various types of microorganisms, including fungi, yeast and algae (eukaryotic), have been proposed as organisms linked to MIC, the major participants in MIC have been proposed to be bacteria and archaea (prokaryotic; Liu et al., 2012). These microorganisms form a biofilm by producing secondary metabolites including extracellular polymeric substances (Procópio, 2020). They can induce or accelerate the corrosion process either by direct metabolic coupling and electron exchange (electrical microbially influenced corrosion – EMIC) or by the generation of reactive metabolites which enhance the corrosion process (chemical microbially influenced corrosion – CMIC (Enning & Garrelfs, 2014; Jeffrey & Melchers, 2003)). Diverse physiological groups including sulfate‐reducing bacteria (SRB), nitrate‐reducing bacteria, thiosulfate‐reducing bacteria, acetogens, methanogens, Fe(II) oxidizers, Fe(III) reducers, and fermenting bacteria have all been reported as microorganisms associated with MIC (e.g. Kip & Van Veen, 2015; Li et al., 2022; Liang et al., 2014; Mand et al., 2014; Videla et al., 2008).

The low water zone in tidal environments is an area of enhanced corrosion rate even without the action of microbes (Jeffrey & Melchers, 2009). Metals and alloys when immersed in seawater can act as an electrochemical cell in an oxic environment where oxygen reduction occurs at the cathode (sub‐aerial) and metal oxidation at the anode (submerged). This effect means that the low water zone in tidal environments is an area of enhanced corrosion rate even without the action of microbes (Jeffrey & Melchers, 2009). The metal oxide thus formed prevents the electron flow between the electrodes creating an open circuit potential (OCP). Microbial ennoblement will increase the OCP to a range nearer to corrosion potential, initiating the process. The mechanism of ennoblement is still not clear (Gümpel et al., 2006; Liu et al., 2012; Trigodet et al., 2019). The presence of anaerobic niches within the biofilms developed on the metal accounts for the presence of anaerobic bacteria in natural biofilms found in oxygenated environments (Beech, 2003). In anoxic conditions, the release of hydrogen will be the cathodic reaction. Even though the electron transfer hypothesis of EMIC is currently accepted as the explanation of MIC, it does not consider the role of microbial enzymes promoting cathodic reactions which are not currently well explained. The classical theory of SRB‐induced biocorrosion describes the electrons from the metal surface entering the bacterial sulfate reduction pathway through a hydrogen intermediate, which is facilitated by the hydrogenase enzyme, sulfur reductase. The difference in the extent of biocorrosion by SRBs because of difference in metabolic specificity is poorly studied. Different species belonging to the same genus can differ in the mechanism by which they contribute to metal corrosion (Beech & Sunner, 2004). Therefore, it is possible that multiple species are involved in the process, through metabolic synergy, and to fully understand MIC in natural environments, the role of consortia of organisms needs to be evaluated.

In addition to direct electron transfer mechanisms, biogenic sulfuric acid (BSA) corrosion caused by the combined action of SRBs and SOBs has been reported as an aggressive form of MIC in both steel and concrete (e.g., Gehrke & Sand, 2003; Uygunoglu & Gunes, 2015).The involvement of both sulfate‐reducing and sulfur‐oxidizing bacteria in accelerated low water corrosion (ALWC – Gubner, 1998; Malard et al., 2013) in marine and estuarine steel structures has been previously recognized (Beech & Campbell, 2008; Beech & Sunner, 2004; Marty et al., 2014; Phan et al., 2020; Smith et al., 2019; Usher et al., 2014). BSA is a multi‐stage process in which SRBs reduce oxidized sulfur compounds, mainly sulfate from seawater, to H_2_S, and SOBs (initially the neutrophilic SOBs such as *Thiobacillus* sp. and *Thiomonas* sp. and then the acidophilic SOBs, eg., *Acidithiobacillus thiooxidans*) produce H_2_SO_4_ (Huber et al., 2016). Sulfuric acid in contact with carbon steel is associated with the corrosion process (Panossian et al., 2012).

Formation and transformation of iron compounds occurs on metal surfaces due to MIC and the corrosion products can be diagnostic of particular corrosion mechanisms (Smith et al., 2024). The major Fe compounds in microbial corrosion systems comprise Green rusts (sulfate green rust – Fe^II^_4_Fe^III^_2_OH_12_SO_4_·yH_2_O), magnetite (Fe_3_O_4_), goethite (α‐FeOOH), lepidocrocite (ϒ‐FeOOH), akaganeite (β‐FeOOH), pyrite (FeS_2_) and mackinawite (FeS_m_) (Smith et al., 2019, 2024; Veneranda et al., 2018). Mackinawite can transform to pyrite (FeS_2_) and Greigite (Fe_3_S_4_) (Zhou et al., 2017). Though analytical techniques such as X‐ray diffraction and Mossbauer spectroscopy have been widely used to determine the relative concentrations of corrosion by‐products, the use of Fourier‐transform infrared (FTIR) or Raman spectroscopy (Pineau et al., 2008) provide fuller (albeit complementary in the case of crystalline products with multiple structures) identification of corrosion products since they avoid issues of autofluorescence of the sample and are applicable to amorphous as well as crystalline material.

**FIGURE 1:**
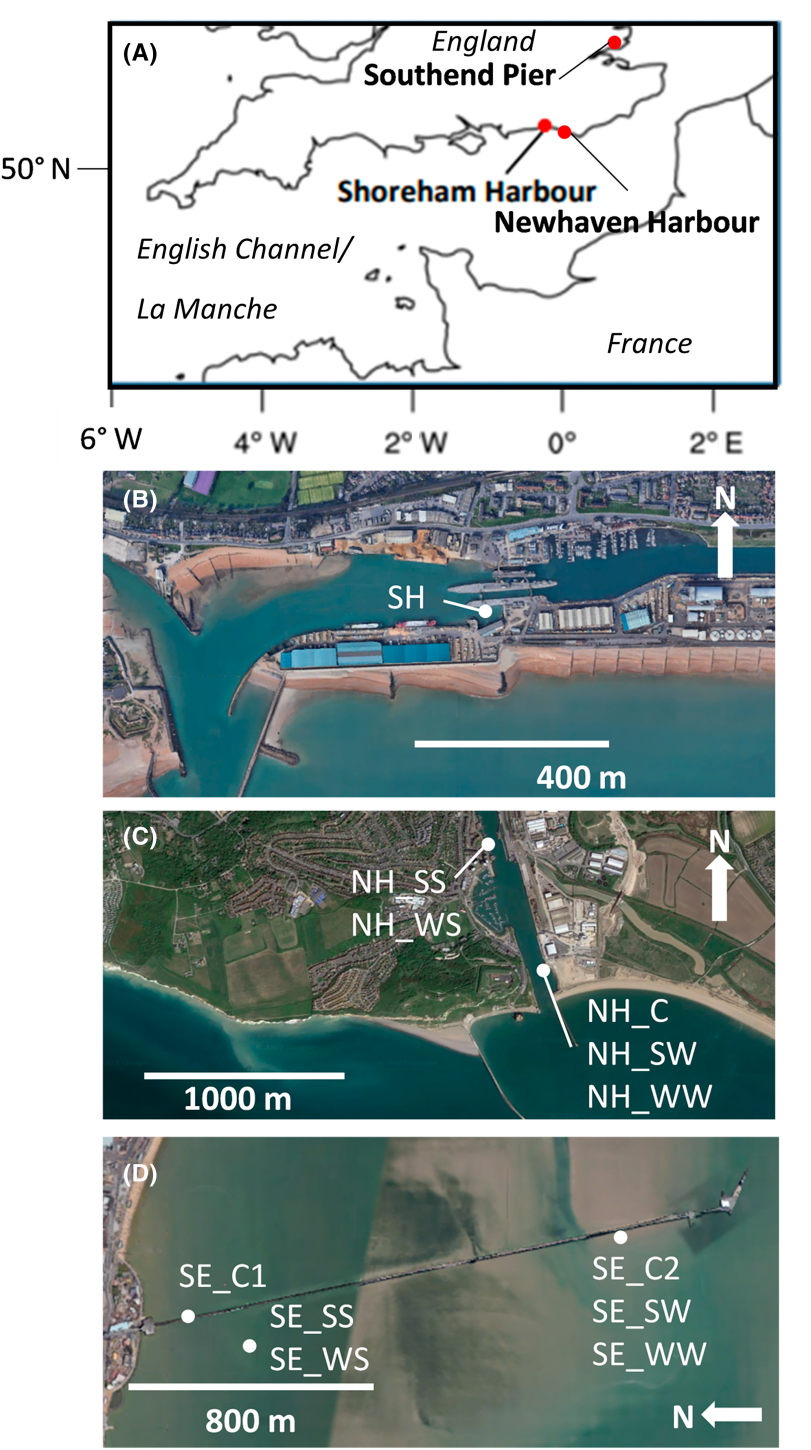
Location of study and sampling sites. (A) Map showing UK sampling sites. (B) Shoreham Port (SH). All samples were collected from a single site. Newhaven Port (NH). Water and corrosion samples were collected from the east side of the port. Sediment cores were collected from the east side because of low tide mud flat exposure. (C) Southend‐on‐Sea Pier (SE). Satellite images from GoogleEarth. C, corrosion products; SS, Summer sediment; SW, Summer water; WS, Winter sediment; WW, Winter water.

In this study, we analysed corrosion blisters from inter‐ and super‐tidal sites, upper and lower sediment core fractions and surface and deeper waters from three different UK coastal sites (Figure 1) to examine the difference in microbial community structure using Illumina‐Miseq sequencing. Previous studies have suggested bed sediment is not a source of causative communities for ALWC (Phan et al., 2019), but similar microbial communities have been reported from steel corrosion and local bed sediment (Beech & Sunner, 2004; Smith et al., 2019). The objective of this study was to identify the microbial communities implicated in corrosion, link these to inferred corrosion mechanisms, and compare the diversity and make up of those communities with those of surrounding water and sediment to determine the source of corrosion implicated organisms. The make up of microbial communities was determined using high‐throughput amplicon sequencing. The different variants of dissimilatory sulfite reductase subunit B gene (*dsr*B) in the population belonging to different sites were determined using high‐resolution melt curve analysis (HRM) in order to expand the sample set utilized and to test the feasibility of the technique for rapid diagnostic tests for sulfate‐reducing prokaryotes implicated in MIC. The sulfur reductase *dsr*B gene variants in SRBs can indicate differences in their activity during MIC, leading to the formation of various corrosion by‐products on metal surfaces, althoughthis can only be confirmed after additional study. The key geochemical parameters of the sea water samples and sediment cores from the vicinity of corrosion sites were also determined to infer environmental controls on microbial distribution. The mineralogy of corrosion blisters was studied using FTIR in order to infer the chemical corrosion mechanism and link this to microbial activity. The difference in microbial community composition and the resulting difference in their metabolic activities leading to varied corrosion products, along with the detection of different variants of *dsr*B genes in the population, could explain the different mechanisms of corrosion occurring under different environmental conditions.

## SITE DESCRIPTIONS

*Shoreham Port*, is an active commercial port facility situated at the mouth of the River Adur in West Sussex, UK (Figure 1B). The harbour bed sediment is dominantly of silt grade quartz and calcite, with kaolinite and illite clays and a significant organic content. Historically, the port also hosted a coal fired power station, and harbour sediments have significant contribution from this, now discontinued, activity. This is dominantly in the form of coal dust, leading to a high organic carbon content. All berthing areas within the port are constructed in an identical fashion: vertically driven carbon steel piling forms the quay face, capped in concrete, with levelled rubble infill and tarmac top layer behind. Estimates of the tidal and non‐tidal perimeter indicate the presence of approximately 6 km of steel piling. The piling is a mixture of both U‐profile and Z‐profile types. The age of piling installed prior to 2005 is not known, but where records exist, some sections of the piling wall can be dated back to the early 20th century. Areas of corrosion consistent with the physical appearance of ALWC (Beech & Campbell, 2008; Klenam et al., 2021) were initially noted prior to 2011. ALWC has been observed in all three principal areas under port jurisdiction (Rousell, 2012). The sampling location lies within the tidal section of the port, adjacent to the main locks, known as the Dredger Berth (50°49′46″ N; 0°14′20″ W). Access to the piling face and sediment bed was via a permanently moored floating jetty.

*Newhaven Port*, is an active commercial and passenger port facility on the mouth of the River Ouse, West Sussex, UK (Figure 1C). Bed sediment is very similar to Shoreham, but without the significant anthropogenic carbon input. The port area has at least 3 km of steel pile wall with a similar construction to Shoreham. The sampling location was adjacent to the site office at 50°47′02″ N, 0°03′27″ E, within the intertidal zone accessible at low tide. Sampling was carried out from the port pilot boat.

*Southend‐on‐Sea pier* is a pleasure pier that now has heritage status as a grade II listed building (iron and steel construction dates to 1889) in the estuary of the River Thames, Essex, UK (Figure 1D). It is the longest pleasure pier in the world. The pier construction consists of hardwood decking on iron piles, extending 2.16 km into the estuary. Sections of iron and steel construction include permanently submerged piles, intertidal piles and iron‐ and steelwork above high tide in the permanently sub‐aerial zone. The latter is still affected by sea water spray. The bed sediment of the site is part of the Thames estuary ‘sea reach’, dominated by bed load transport from the fully marine environment (Prentice & Gray, 1972). Sediment is of fine sand grade with siliclastic and bioclastic components and a lower clay content than the other 2 sites. The Holocene deposits are relative thin (~15 cm from coring as part of this study) compared to both Shoreham and Newhaven (several m) and subject to active scour (Sturt & Dix, 2009). The Holocene sediments are underlain by London Clay. There are potential nutrient contaminant inputs from storm and sewage sludge effluent (Baugh et al., 2013). Sampling was conducted form the pier itself, or from low tide access below the pier.

### Sample description

Sediment cores up to 70 cm in length and 2 cm in diameter were taken using a drop corer fitted with an internal plastic sleeve during three seasons: Spring, Summer and Winter in 2021–2022. The plastic sleeve meant that cores could be taken without exposing sub‐surface sediment to the atmosphere. Two cores were taken at each site – one for geochemical analysis and one for DNA extraction. The cores for microbiological analysis were transferred to a freezer and stored at −80°C until processing.

Seawater samples were collected using a polytetrafluoroethylene (PTFE) bailer on a Teflon‐coated steel cable. The bailers consist of a 1 m PTFE tube, open at the top with a ball valve at the base, allowing water to flow through during lowering, but not during raising. This allowed for depth constrained water samples to be collected at 1 m integrated intervals. The samples to be used for DNA extraction were stored at −80°C, and the duplicates were used to study water chemical parameters.

**FIGURE 2:**
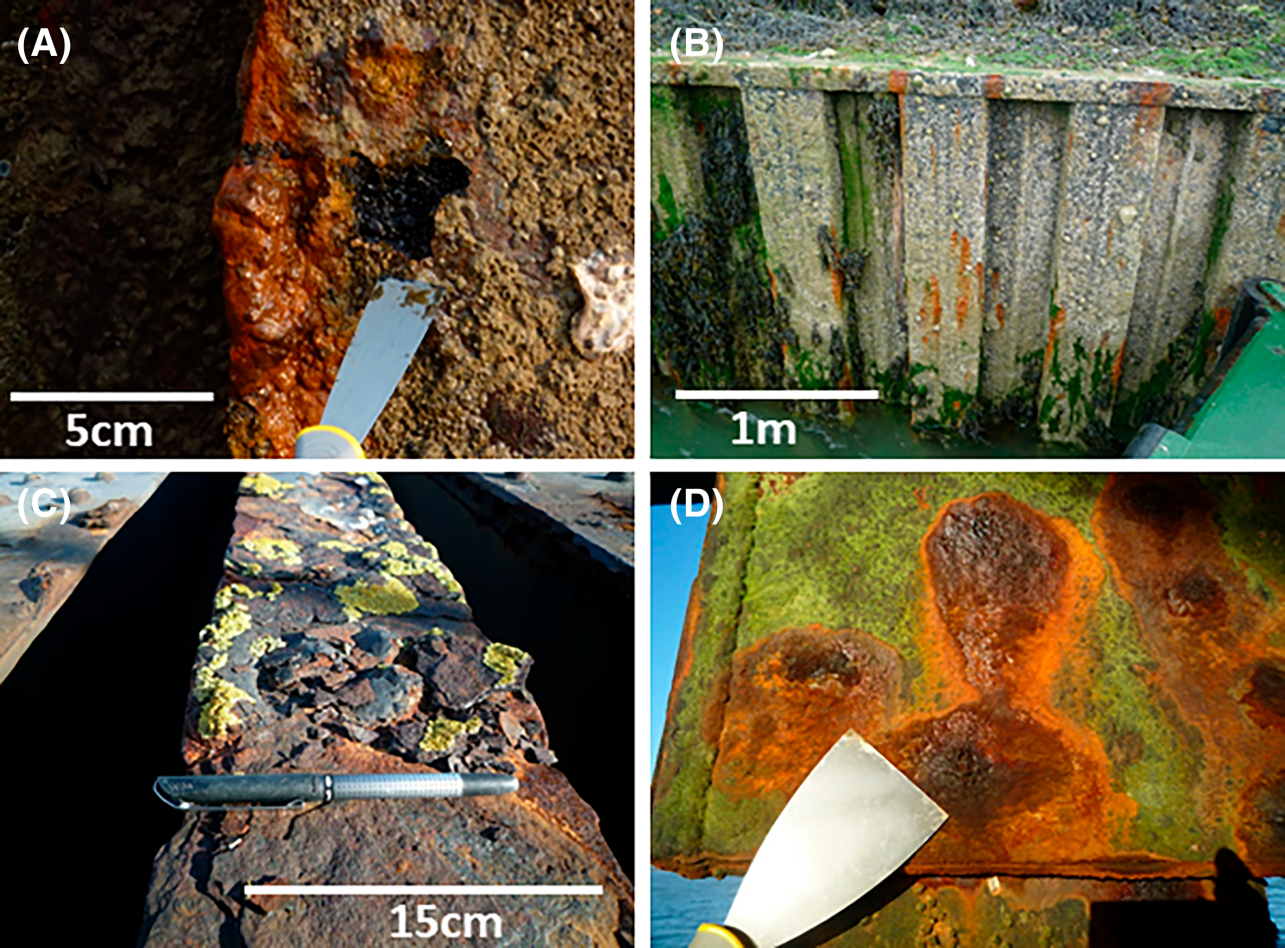
Examples of corrosion types developed at the study sites. (A) MIC corrosion blister (ALWC) with small area removed showing sulfidic blister contents overlying acid etched steel. Intertidal steel piling, Shoreham Port. (B) MIC corrosion blisters developed on intertidal steel pile walling, Newhaven Port. (C) Sub‐aerial, splash zone corrosion developed on steel beam, Southend‐on‐Sea Pier. (D) Intertidal corrosion blisters developed on steel beams, Southend‐on‐Sea Pier.

Corrosion samples were collected from piling surfaces using stainless steel scrapers or scalpels pre‐cleaned with distilled water. Samples were immediately placed into zip lock plastic bags and then into sealed plastic boxes with Thermo Scientific Anaerogen™ 2.5 L sodium meta‐bisulfite sachets to preserve anoxic conditions from the interior of corrosion blisters, and to prevent further oxidation of corrosion products. At Shoreham and Newhaven samples were taken from the intertidal zone immediately above low water (Figure 2A,B). At Southend one sample was taken from a subaerial steel beam showing lichen growth for comparison with low water samples (SE_C1; Figure 2C). Other samples were taken from intertidal and splash zone corrosion (Figure 2D). Intertidal corrosion samples at Southend did not return enough DNA for high‐throughput amplicon sequencing, but were included in the sample set used for HRM. As a result, a sample from the splash zone was selected which had produced a high enough quality sample to be used (SE_C2).

## METHODS

### High‐throughput amplicon sequencing, processing of raw reads and statistical analysis

Frozen sediment cores were scraped to remove the smeared outer layer and the interior sectioned into 2–4 cm intervals using a steel palette knife which was sterilized with ethanol after each sectioning. The inner portion from each section (0.6 g) was used for DNA extraction. Sea water samples were allowed to thaw to room temperature and 180 mL was filtered through 0.22 μm pore size membrane filter (MF‐Millipore™ Membrane Filter) which was then used for DNA extraction. The samples collected during the summer and winter seasons were used for metagenomic study to limit the number of samples to be analysed and processed. The frozen corrosion samples were allowed to dry overnight in a vacuum desiccator with Na‐metabisulfite to prevent oxidation. The corrosion samples were then ground using a sterilized mortar and pestle.

Genomic DNA was extracted from each corrosion and sediment sample using Qiagen DNeasy PowerSoil Pro Kit and DNeasy PowerWater Kit (MoBio PowerSoil kit before acquisition by Qiagen) with slight modifications to the manufacturer's recommended protocol by doubling the amount of initial sample for the analysis and including a heating step (60–65°C for 10 min.) to ease the mechanical disruption of cells. The concentration of DNA was estimated using a Qubit™ 3.0 Fluorometer (Thermo Fischer Scientific). The extracted DNA was stored at −80°C prior to further analyses.

An Illumina MiSeq Personal Sequencer was used at EUROFINS genomics, Germany, to determine the community structure, richness, and diversity of total bacteria present in the samples based on 16S rRNA gene fragments (V1‐V3 region; ~300 bp) amplified using the primer pairs 341F/806R. Paired‐end reads were assigned to the samples based on their unique barcode. The raw reads were truncated by removing the barcode and primer sequence using FLASH (Version 1.2.7) (Magoč & Salzberg, 2011).

The raw reads were then quality‐trimmed using Cutadapt ver.3.4 (Martin, 2011) and further processing of demultiplexed sequence reads were done using the “DADA2” (Callahan et al., 2016) and “DECIPHER” (Wright et al., 2012) packages in R software (version 4.0.5) (R Core Team, 2013). In brief, the sequences were quality filtered, trimmed and assembled. For trimming the forward and reverse reads, lengths of 200 bp and 250 bp, respectively, were chosen based on the quality profile, maintaining a median quality of 30 or above and the maximum number of expected errors in the reads were set to 2. The forward and reverse reads with a minimum overlap of 20 bp were merged, and the chimeras were removed using DADA2's inbuilt function. Amplicon sequence variants (ASVs) generated were taxonomically classified using the RDP naive Bayesian classifier (Wang et al., 2007), trained with the Silva 16S rRNA gene taxonomic training data formatted for the ‘DECIPHER’ package (Silva version‐138.1). The data analysis of the taxonomy assigned ASVs were done using the packages, ‘phyloseq’ (McMurdie & Holmes, 2013), ‘DESeq2’ (Love et al., 2014) and ‘vegan’ (Jari Oksanen et al., 2018) and the visualization using ‘ggplot2’ (Wickham et al., 2016) and ‘ComplexHeatmap’ (Gu et al., 2016) in R. neighbour‐joining phylogenetic trees were created using the plot_tree function in the ‘phyloseq’ package.

### High‐resolution melt curve analysis of dissimilatory sulfite reductase subunit B gene ( *dsr*B) genes in samples

High‐resolution melt curve analysis (HRM) is employed for rapid detection of sequence variants. This method has replaced the traditional polymerase chain reaction (PCR), and sequencing of PCR amplicons as the technique is rapid, highly sensitive, analysis can be done in real time, and it eliminates the need for electrophoretic analysis after traditional PCR reaction for identification (Winder et al., 2011). The nucleotide sequence, amplicon length and GC/AT ratio are the key factors determining the amplicon melt temperature profile (Ririe et al., 1997; Varga & James, 2006). qPCR instruments with precise temperature control and fluorescence detection are available to detect PCR amplicons with single nucleotide polymorphisms (SNPs) (Vossen et al., 2009). We have investigated the application of HRM in order to identify *dsr*B gene (used widely for the identification of SRBs) variants using a rapid, highly sensitive qPCR‐HRM method. The capacity to differentiate between gene variants through melt peak analysis using standard qPCR equipment has been illustrated in this study. The configuration of the melt peak(s) (referred to as the melt profile) and their respective temperatures were both found to offer significant diagnostic insights. The concentration of extracted DNA from the various sources was normalized to 5 ng/μL. Existing primer pairs previously reported for the detection of *dsr*B gene were used in this study, *DSR*p2060F (CAACATCGTYCAYACCCAGGG) and *DSR*4R (GTGTAGCAGTTACCGCA) ~ 350 bp (Kaya et al., 2017). Briefly, this gene was amplified and melt curves generated using a Qiagen Rotor‐Gene Q real‐time PCR cycler. The PCR mix consisted of 10 μL of 2× Type‐it HRM PCR Master Mix (Qiagen), 2 μL of each primer at a concentration of 10 μM, 1 μL (5 ng/μL) of template DNA and Qiagen RNase‐free water to a total volume of 20 μL. The PCR amplification cycles and temperatures used are presented in Table 1. Data were acquired and analysed with the accompanying Rotor‐Gene Q Software. Normalized melting curves of test samples along with a non‐template control (NTC) were compared.

**TABLE 1:** The optimum cycling number and temperatures standardized for amplifying *dsr*B genes in corrosion, sediment and seawater samples.

Cycle	Cycle point
Hold	Hold @ 95°C, 5 min 0 s
Cycling (60 repeats)	Step 1: Hold @ 95°C, 10s
	Step 2: Hold @ 55°C, 30s
	Step 3: Hold @ 72°C, 20s
Melt	Ramp from 80°C to 90°C
	Hold for 90s on the 1st step
	Hold for 2 s on next steps

### Characterization of corrosion by‐products using FTIR

The frozen corrosion samples were allowed to dry overnight in a vacuum desiccator with Na‐metabisulfite. The corrosion samples were then ground using a sterilized mortar and pestle. Fourier transform infrared spectroscopic (FTIR) measurements were performed using a Perkin Elmer, Spectrum 65 spectrometer, fitted with an attenuated total reflectance accessory employing a ZnSe crystal. The samples were measured in the spectral range 4000–600 cm^−1^ at a resolution of 4 cm^−1^. Each spectrum was collected from 16 scans. The data were analysed using PeakFit version 4.12 (Jandel Scientific Software) to convert data from transmission to absorbance mode and to allow deconvolution of absorbance peaks. Analysis was focussed in the range 650–1600 cm^−1^.

### Estuarine and pore water analysis

Water samples were analysed on site, immediately after collection, for pH, dissolved oxygen, electrical conductivity, temperature and redox potential using an Aquaread AP2000 multi parameter probe, calibrated prior to each day of deployment. Duplicate depth constrained water samples were analysed using ion chromatography (IC) for major anions using a Dionex ICS‐1100 IC with an Ion Pac AS23 column following sample dilution 10 times with 18mOhm distilled water to reduce the scale of the chloride peak and allow resolution of other anions. Sediment cores collected parallel to those used for microbial analysis were used to extract pore water using Rhizon samplers (Seeberg‐Elverfeldt et al., 2005) using pre‐drilled holes in the plastic sample sleeve. The holes were covered with duct tape during sampling and storage. All the samples to be analysed were filtered using a 0.45 μm pore size membrane filter (MF‐Millipore™ Membrane Filter).

## RESULTS

### Microbial diversity and community composition

**FIGURE 3:**
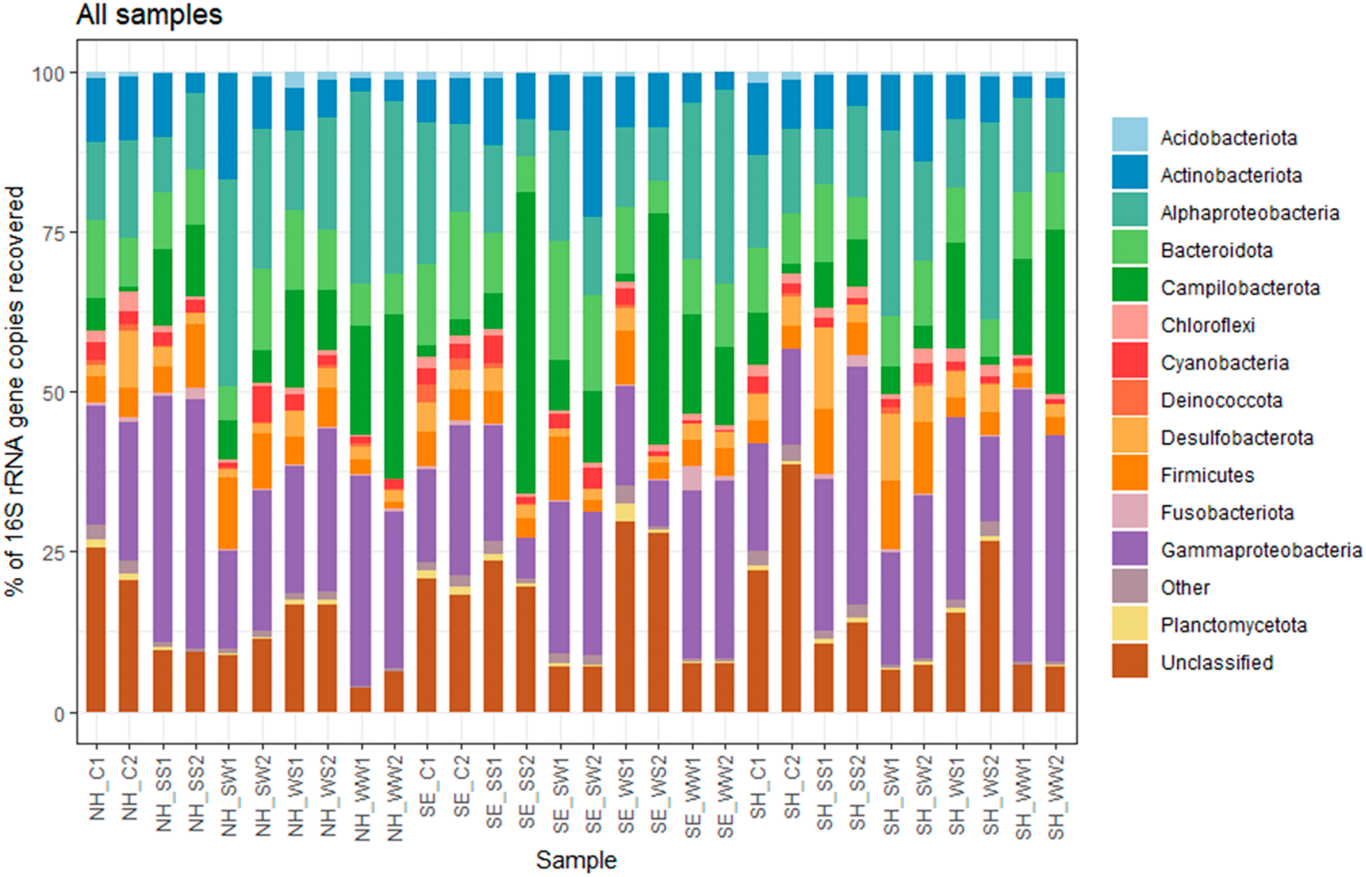
16S rRNA gene‐based relative abundance of dominant bacterial groups (relative abundance >1%) observed in all samples collected from Newhaven (NH), Southend (SE) and Shoreham (SH). NH_SS1, Newhaven surface sediment (summer); NH_SS2, Newhaven deep sediment (summer); NH_WS1, Newhaven surface sediment (winter); NH_WS2, Newhaven deep sediment (winter); SE_SS1, Southend surface sediment (summer); SE_SS2, Southend deep sediment (summer); SE_WS1, Southend surface sediment (winter); SE_WS2, Southend deep sediment (winter); SH_SS1, Shoreham surface sediment (summer); SH_SS2, Shoreham deep sediment (summer); SH_WS1, Shoreham surface sediment (winter); SH_WS2, Shoreham deep sediment (winter).

**FIGURE 4:**
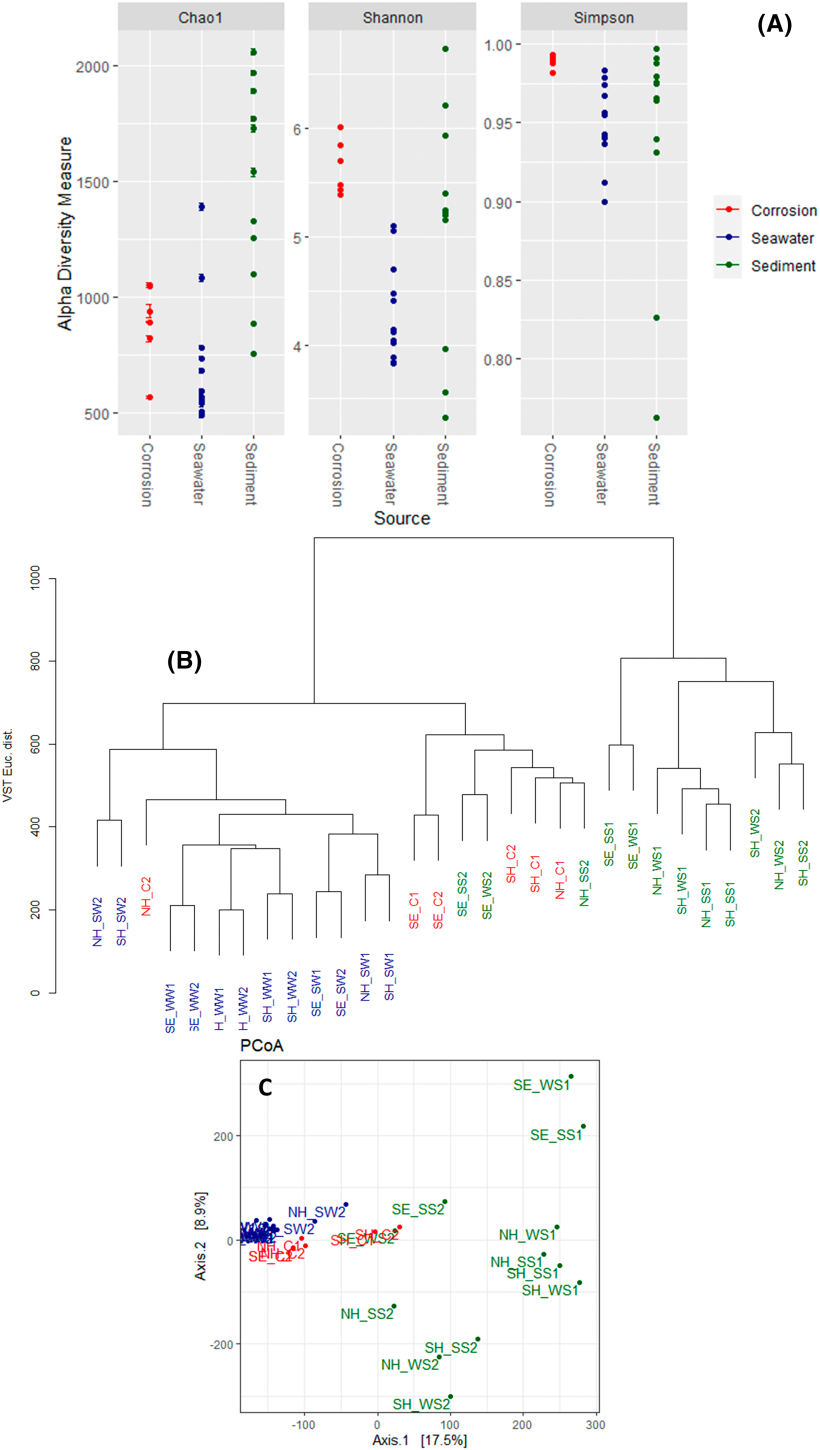
(A) Alpha diversity measures calculated for all the samples from 3 different sample types. (B) Dendrogram and (C) principal coordinates analysis (PCoA) of weighted UniFrac distances showing bacterial community similarity in all the samples. Samples were grouped into three categories, indicated by colours, based on sampling sources (Red = corrosion samples, Blue = seawater samples, Green = marine sediment samples).

High‐throughput amplicon sequencing of 16S rRNA gene amplicon libraries from 30 samples representing discrete sea water and marine sediment depths (surface and ~50 cm deep) in two different seasons from each of the three sites (summer and winter) and two corrosion blisters from each site produced a total of 3,412,913 bacterial raw reads, which after removal of low‐quality and chimeric sequences provided 2,504,836 reads (Supplementary results‐RawReads_perc; NCBI SRA Bioproject ID: PRJNA974398). A total of 52 bacterial phyla were identified across all the samples analysed. The dominant phyla in all samples that exhibited a relative abundance of >1% in at least one sample are shown in Figure 3 (and Supplementary results‐Phylum%, Phyla>1%). These phyla accounted for more than 97% of the total sequences in each sample. Proteobacteria (Alphaproteobacteria and Gammaproteobacteria together constituting 40.4%) were the most abundant phyla and accounted for 31%–63% in Newhaven, 31%–58% in Southend and 31%–47% in Shoreham. The corrosion samples in all the sites were found to possess 28%–37% of Proteobacterota (Figure 4).

The second most abundant phylum was Campilobacterota, which consisted of about 11.3% in total. This phylum was the predominant one at Southend up to a proportion of 47.1% compared to the other two sites with a maximum of 25%. In Southend, the deep sediments collected during summer and winter had the highest proportion (36.07%–47.1%), whereas the deep sediments from other sites had 7%–11% (summer) and 9.4% and 1.4% in Newhaven and Shoreham correspondingly during winter. In seawater samples, the ones collected during summer (4%–11%) had a lower percentage of Campilobacterota than those from winter season (12%–25%) in all the three sites. About 3.5%–5% Campilobacterota were found in the corrosion samples (Figure 4). Other phyla present and abundant in the samples were Bacteroidota and Actinobacteriota; Actinobacteriota being the lowest proportion in the winter sea water samples from both depths (2%–4.5%) in all the 3 sites. A high proportion of unclassified 16S genes were recovered corresponding to 20%–25% in corrosion samples, 10%–30% in sediment samples and 4%–10% in seawater samples.

The α‐diversity measures (Shannon/Simpson indices and Chao1) indicate that microbial diversity is greater in marine sediments, followed by corrosion samples, compared to seawater (Figure 4A). The rarefaction curves for random sampling generated by subsampling to the depth of the sample with the least number of reads in the dataset (Supplementary results‐Suppl‐Figs.; Figure 1) show major groupings of the samples based on sampling sources. The species accumulation curves showed that the deeper sequencing revealed that the majority of diversity among ASVs and the libraries presented the majority of the bacterial diversity.

The similarities and dissimilarities between the bacterial communities in the different samples were further quantified through clustering analysis based on the weighted UniFrac distance metric (Figure 4B). For all sites the communities were most similar in corrosion and sediment samples, with both strongly distinct from the water samples. The exception is a single corrosion sample from Newhaven, for which the closest comparison was with local seawater. Samples from Southend varied from the other two sites, Newhaven and Shoreham, in all the analysis, in terms of both microbial richness and diversity. A permutational ANOVA‐test supported the sample clustering pattern with a *p*‐value 1.37E‐07.

**FIGURE 5:**
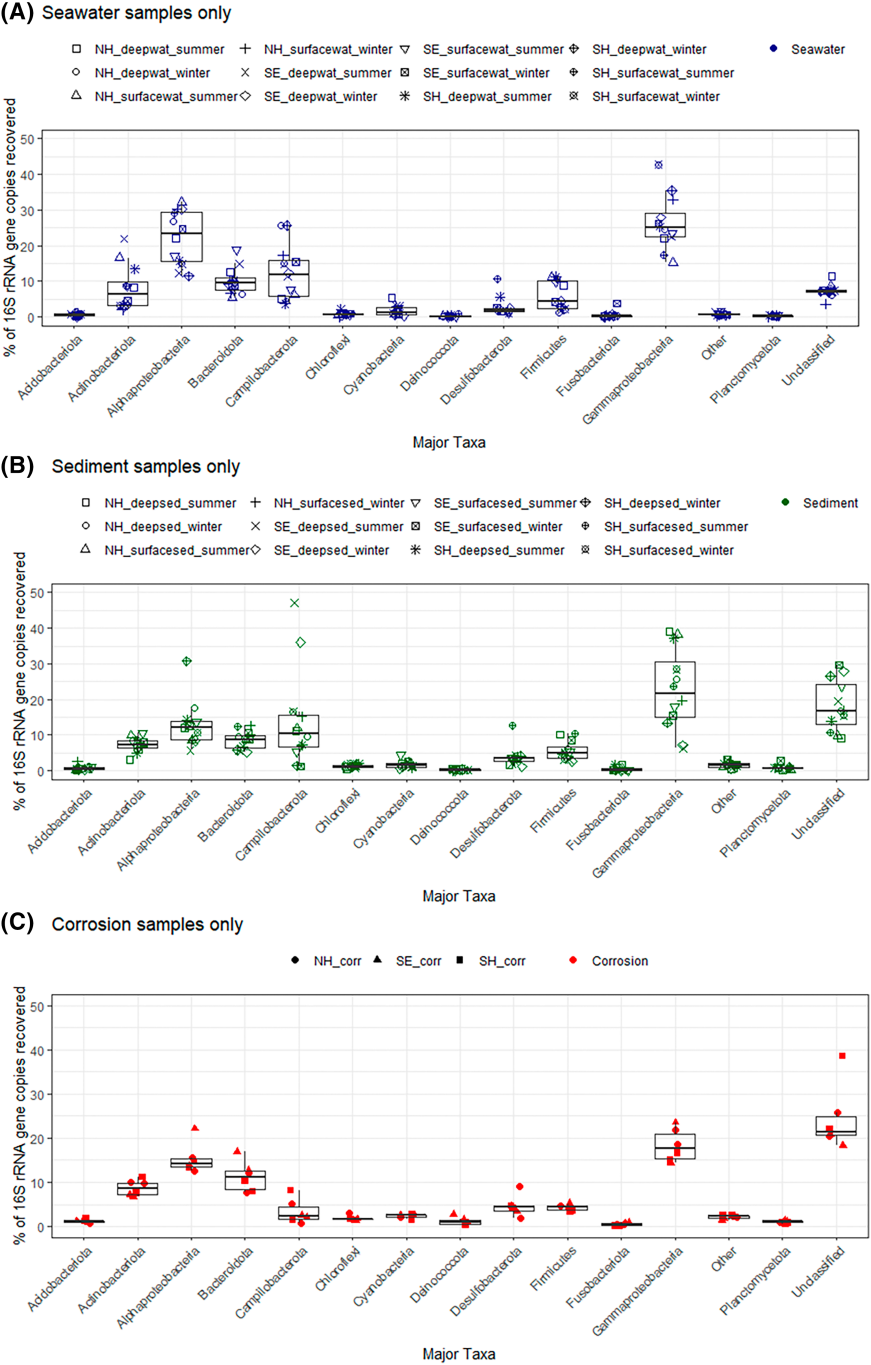
16S rRNA analysis showing the proportion of microbial communities retrieved from three different sample types from two different seasons (seasonal sampling is not applicable to corrosion samples). (A) Water. (B) Sediment. (C) Corrosion.

When microbial community structures of the collected samples (marine water and sediments) were analysed based on dry and wet seasons (summer and winter), there was a significant difference (*p* ≤ 0.05 using betadisper and adonis) between the two seasons suggesting temperature and precipitation as probable key factors determining microbial community structures (Figure 5A–C). In water samples, the total percentage of recovered phyla from actinobacteriota, Bacteroidota and Desulfobacterota were higher during the summer season. However, Campilobacterota and Gammaproteobacteria were in higher abundance during winter season, and least during summer season. In sediment samples, the summer samples were rich in Actinobacteriota, Desulfobacterota, Firmicutes and Gammproteobacteria. Deep sediment samples from Shoreham and Newhaven during summer were found to be rich in Gammaproteobacterota, whereas deep winter samples had a high proportion of Alphaproteobacterota. In Southend, the deep samples from both the seasons had Campilobacterota as the dominant phyla.

**FIGURE 6:**
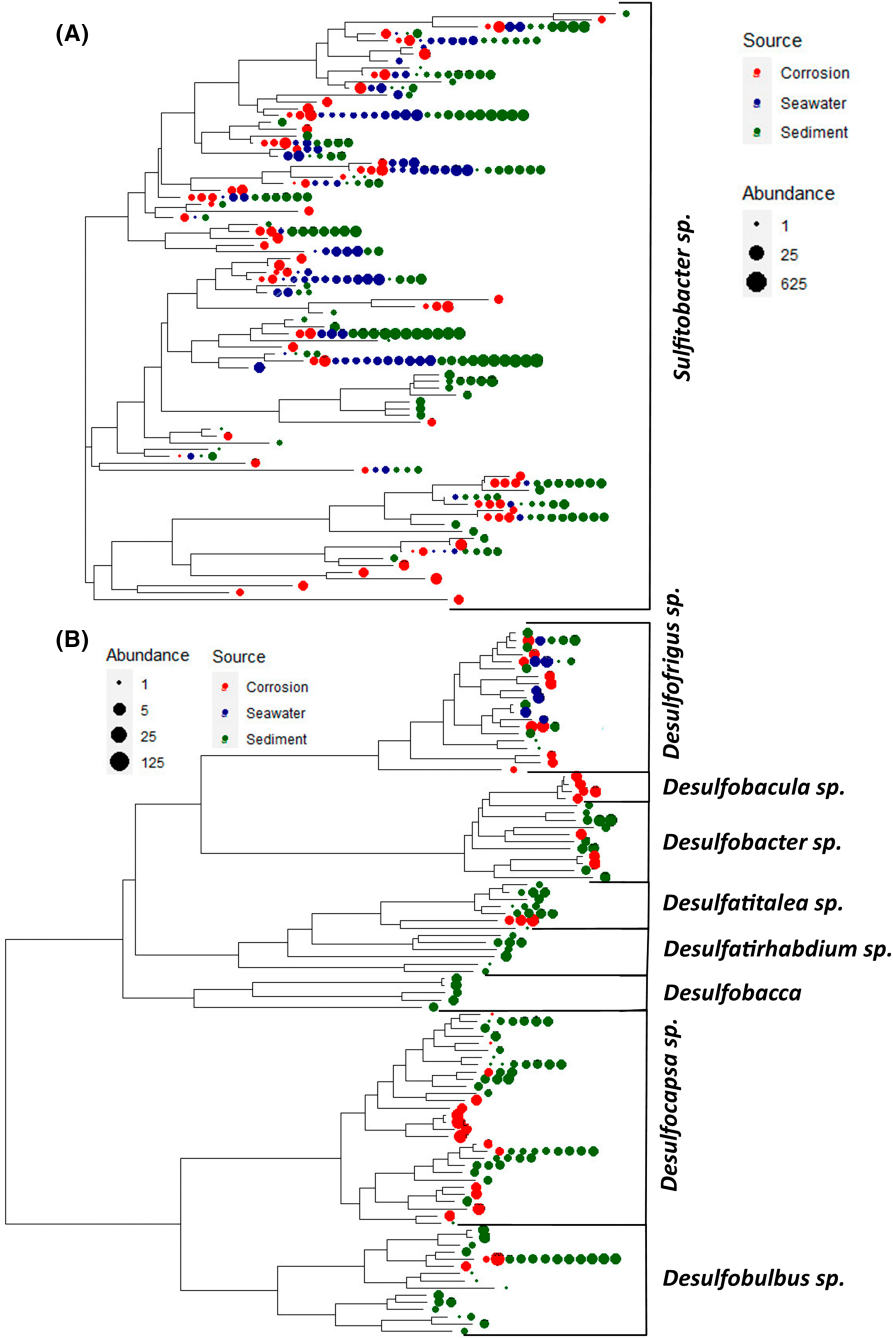
Neighbour‐joining dendrogram of (A) *Sulfitobacter* sp., an S‐oxidizing genera (B) some of the identified S‐reducing genera such as *Desulfofrigus*, *Desulfobacula*, *Desulfatitalea*, *Desulfatirhabdium*, *Desulfobacca*, *Desulfocapsa* and *Desulfobulbus* retrieved from three different sources (corrosion, sediment and seawater) with relative abundance shown by symbol size. The tips of the dendrograms are shaded according to the source in which the particular ASV was observed (Red = corrosion, Blue = seawater and Green = sediment), and the size of the tips shows the abundance which is related to the number of sequencing reads. Other dendrograms of some of the common genera associated with MIC are included in the Supplementary results‐Suppl‐Figs.; Figure 2.

Bacterial composition at the lower hierarchical levels that were reported to be involved in MIC or in S‐cycling was further investigated to reveal potential spatial variations in microbial communities (Figure 6A,B; Supplementary results – Suppl‐Figs.; Figure 2). *Deferrisoma* sp., which is an Fe‐reducing bacterium, was present mostly in sediment samples along with *Zixibacteria* and some members from phylum Desulfobacterota such as *Desulfatiglans* sp., *Sva0081 sediment group* and *SEEP‐SRB1*. *Candidatus electrothrix*, a cable‐bacterium belonging to Desulfobacterota phylum, which is capable of performing electrogenic sulfur oxidation in anoxic environments through long‐distance electron transport, was identified only in Southend sediment samples. The genera from Desulfobacterota that were prominent in corrosion samples included *Desulfofrigus*, *Desulfobacula*, *Desulfocapsa*, *Desulfoconvexum*, *Desulfocarbo* and *Desulfonatronum* and from seawater samples was *Desulfotalea*. *Sulfurimonas* and *Sulfurovum*, belonging to Campylobacterota, dominated as the major sulfur‐oxidizing genera, mainly in sediment samples with the least proportion in sea water and corrosion samples along with a high proportion of unidentified members from Arcobacteraceaea. The presence of Acrobacteraceae belonging to Campilobacterota in Southend can be attributed to the storm water release which occurred during the period (Venâncio et al., 2022). *Colwelia*, *Sulfitobacter*, *Shewanella* and *Ferrimonas* were found to be present in all the three sample sources (Supplementary results – Suppl‐Figs.; Figure 2).

### HRM analysis of *dsr*B genes in the samples

**FIGURE 7:**
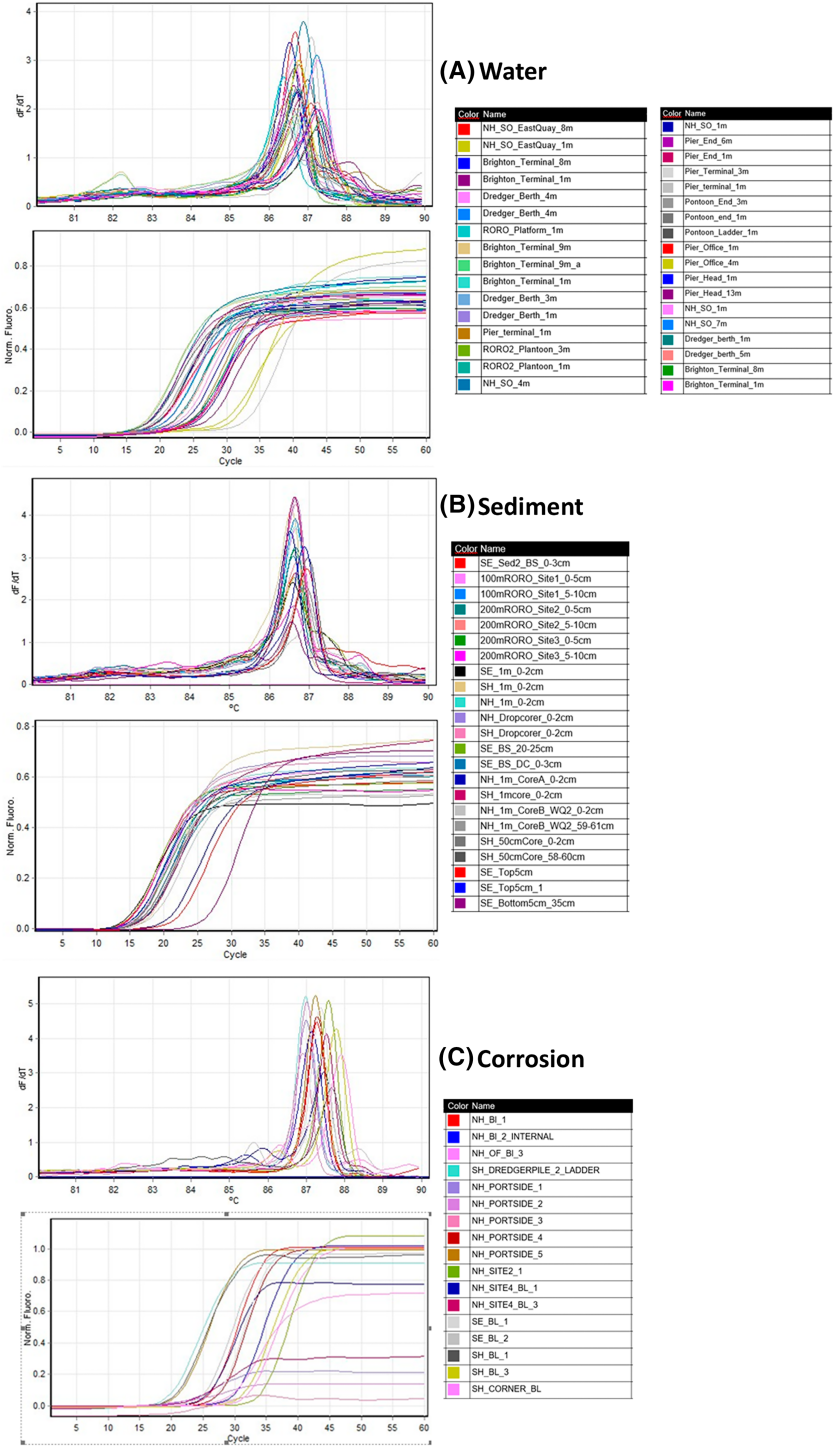
The HRM melt curves and quantitation data obtained for *dsr*B genes from eDNA extracted from three different samples from three different sites. (A) Water. (B) Sediment. (C) Corrosion. The sample details are provided in Supplementary results‐HRM_sampledetails.

**FIGURE 8:**
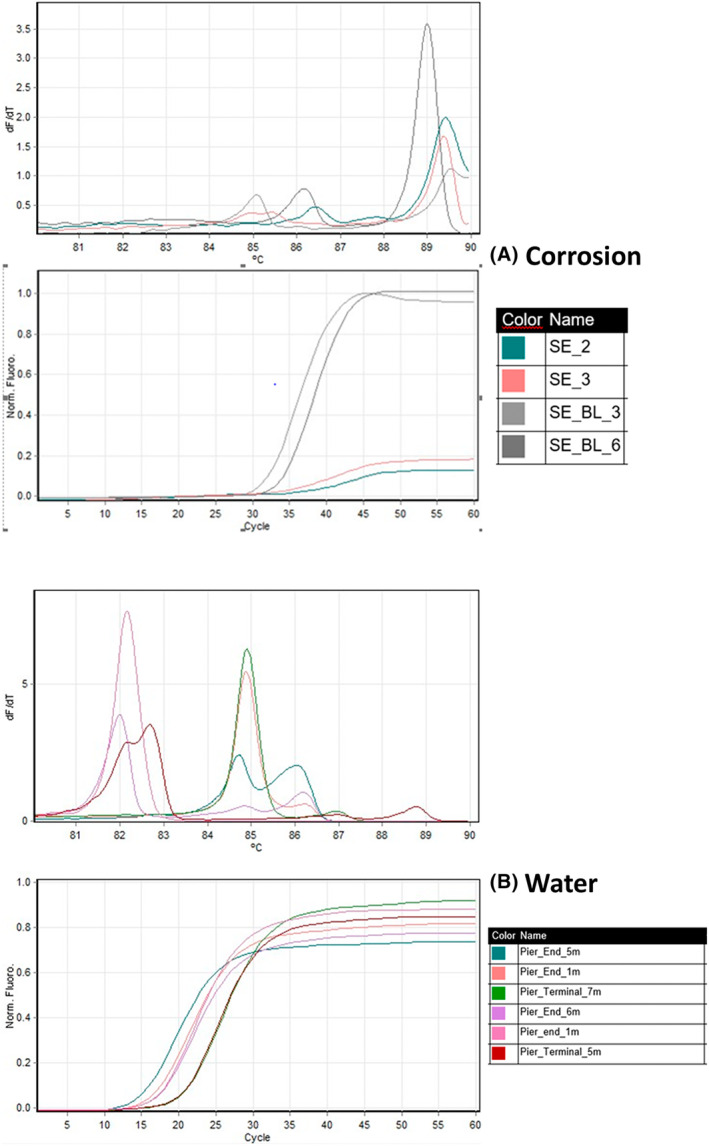
Different variants of *dsr*B genes identified only from Southend site. (A) Corrosion samples. (B) Water samples.

The target gene, *dsr*B (350 bp), melts at a temperature between 86 and 88°C using the standardized protocol developed during the study (Figure 7). The *dsr*B variants can be identified by the shift in melt curve temperature and from the obtained curve. As observed from amplicon analysis results, HRM analysis identified in different variants of the *dsr*B gene in Southend samples (Figure 8). HRM analysis is able to differentiate DNA sequences on the basis of their composition, length, GC content, or strand complementarity. It is anticipated that each variant will produce a unique melting temperature (Tm, which is the temperature at which the DNA duplex will dissociate to single stranded and indicates the duplex stability). The shift in the *dsr*B Tm from 86–88°C to a higher temperature (88–90°C) in the corrosion samples and to 81–86°C in sediment samples from Southend site indicates the presence of SRB species possessing different variants of this gene compared to Shoreham and Newhaven.

### FTIR spectroscopy of corrosion products

**FIGURE 9:**
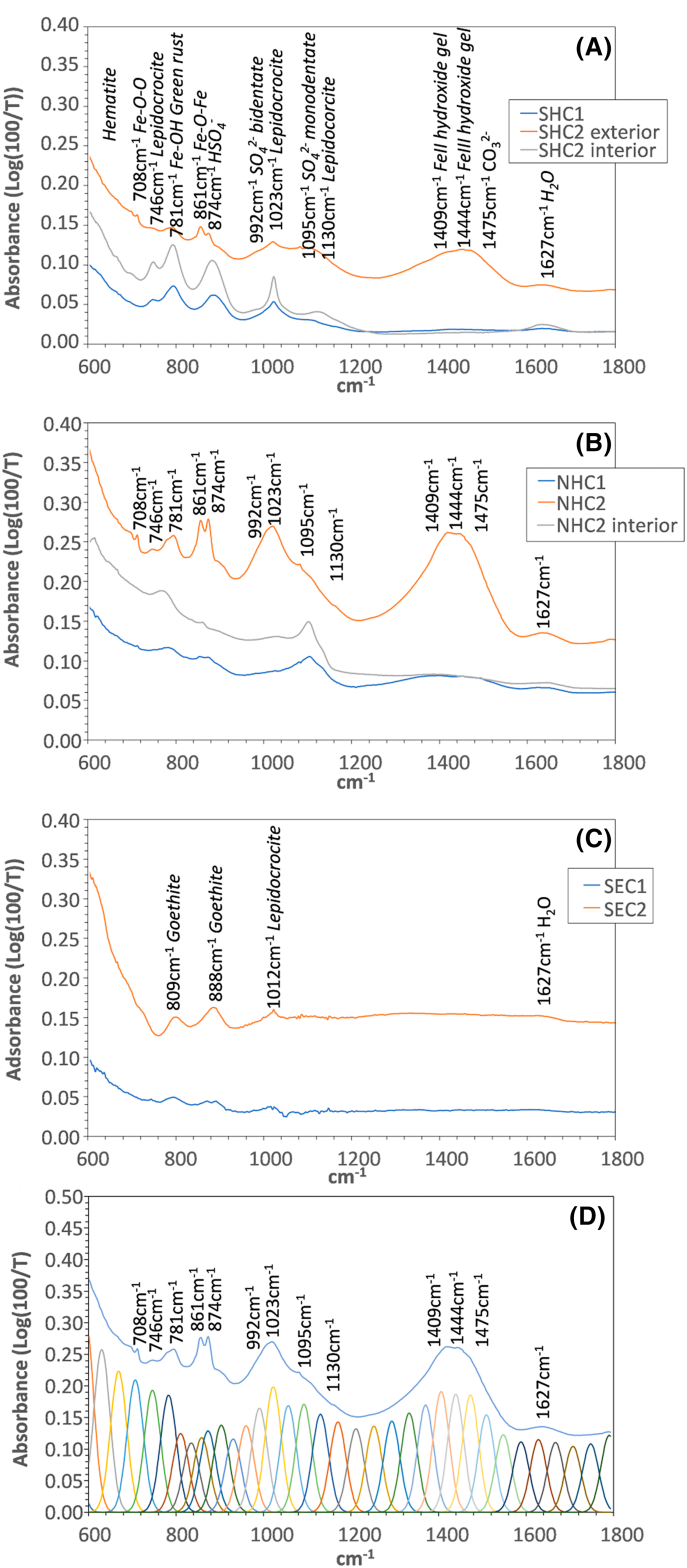
Results of FTIR spectroscopy of corrosion products from (A) Shoreham, (B) Newhaven, (C) Southend. (D) An example of peak deconvolution for accurate determination of peak position using PeakFit 4.12 from sample NHC2.

Figure 9A–C shows the results of FTIR spectroscopy of the corrosion samples used for DNA extraction. Individual peak positions in the range 600–1800 cm^−1^ were constrained using the PeakFit 4.12 software package. Following transformation from % transmission to absorbance (log(100/%T)) and a non‐parametric data filter, peaks were identified using no baseline, Gaussian amplitude deconvolution and a full width at half maximum peak height of 25.4 cm^−1^. The resulting fits were accepted if the *r* ^2^ exceeded 0.99. An example of the resulting peak deconvolution is shown in Figure 9D. The overall form of the spectra (fit with multiple peaks) results from the presence of haematite, with broad scale adsorption from 600 to 800 cm^−1^ (RRUFF database; accessed 10/10/2022). Individual peaks isolated from the broad adsorption pattern are labelled in each spectrum. The prime distinguishing feature between intertidal corrosion samples and splash zone to subaerial samples is the broad amalgamated peak from 1400 to 1500 cm^−1^, the adsorption band from ~950 to 1150 cm^−1^, and isolated peaks at 708, 746, 781, 861 and 874 cm^−1^, all visible at Shoreham and Newhaven. In contrast the samples from Southend show only adsorption peaks at 809, 888 and 1012 cm^−1^.

### Sea water and pore water chemistry

**FIGURE 10:**
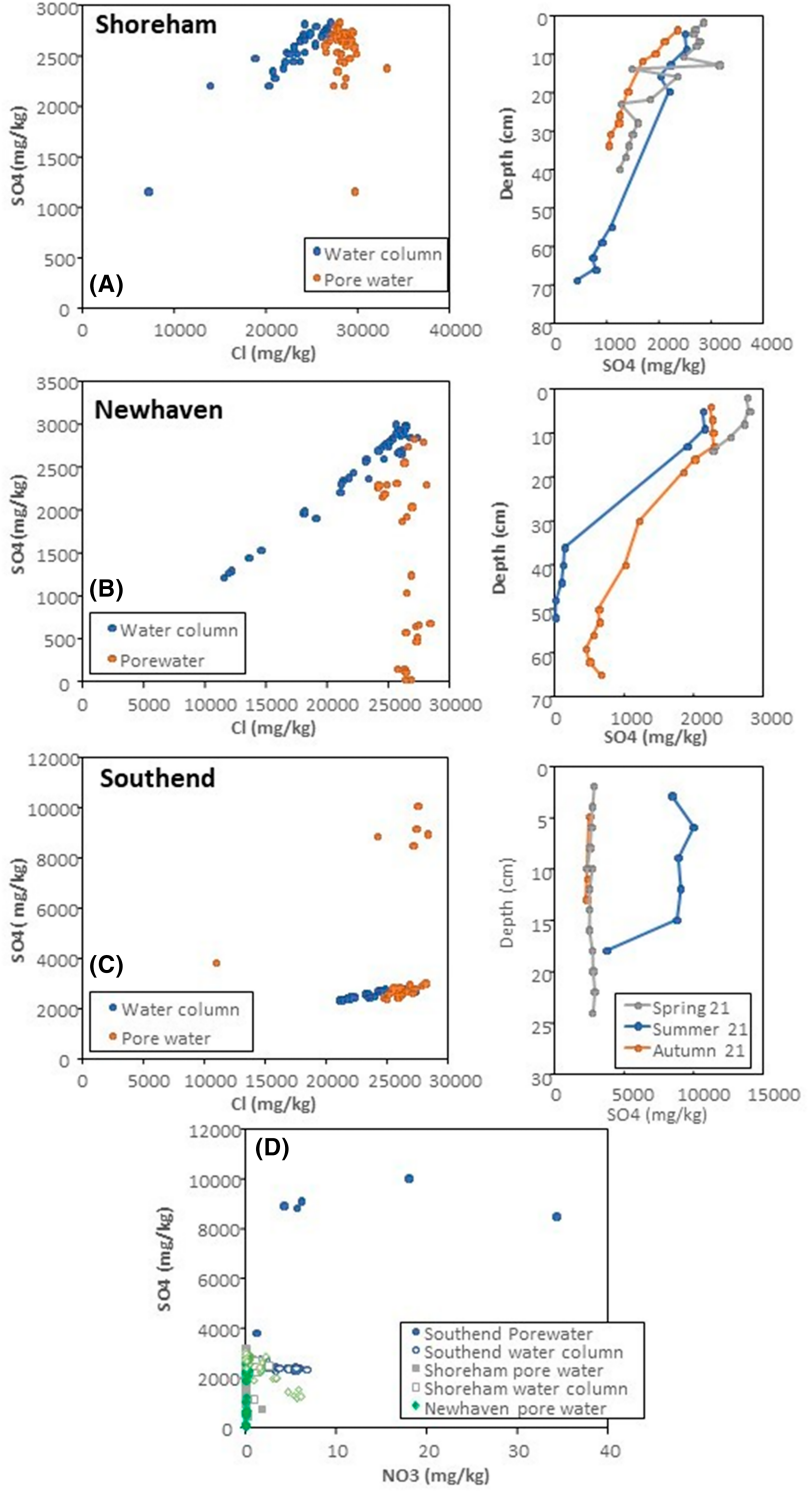
Results of anion analysis by ion chromatography for port water column and bed sediment pore waters. Seasonal sampling periods were in April–May, July and November 2021. (A) Shoreham. (B) Newhaven. (C) Southend. (D) Plot of sulfate against nitrate for all sites.

The results of anion analyses of the port water column and sediment pore fluids are shown in Figure 10. In all three sites, the water column analyses of chloride and sulfate lie on a linear trend with a fixed ratio representing the mixing of seawater with river water within the estuarine environment. Variation in the overall concentration of anions is driven by the tidal cycle. There is little evidence for anthropogenic influence in the port and estuarine water column. Nitrate shows a negative correlation with sulfate in the main water column (Figure 10) indicating that the primary source of nitrate in this context is fluvial runoff. None of main water column analyses indicate significant nutrient contamination. The anion chemistry of sediment pore water shows significant departures from a fixed sulfate‐chloride ratio at Shoreham and Newhaven. Newhaven in particular shows a trend of decreasing sulfate at a relatively restricted range of chloride concentration. The chloride concentration is at the higher end of the surface water range as the water infiltrating into bed sediment is dominated by the denser seawater rather than lower density fresh water. The decline in sulfate concentration can therefore be related to the action of sulfate‐reducing bacteria/archaea in the bed sediment with increasing depth, indicative of bed sediment anoxia (Figure 10). This trend is not apparent at Southend, indicating limited activity of sulfate‐reducing prokaryotes. This indicates relatively well oxygenated bed sediment at this site. The departure from a freshwater–seawater mixing line at this site is to very high sulfate contents, which also correlate with high nitrate concentrations.

The major shift in nitrate and sulfate concentration in porewater samples collected from Southend during the summer season is likely to be attributed to a storm sewer release event which occurred during the period, notably the higher amount of sulfate (approx. 10,000 ppm) (Figure 10). A high proportion of Arcobacteraceaea in the sediments of Southend samples further confirms this finding. The presence of high amount of Acrobacters in environmental water bodies has been suggested to be due to faecal pollution, although several species have been described as native to marine environments (Collado et al., 2008; Fong et al., 2007; Salas‐Massó et al., 2018; Venâncio et al., 2022).

**FIGURE 11:**
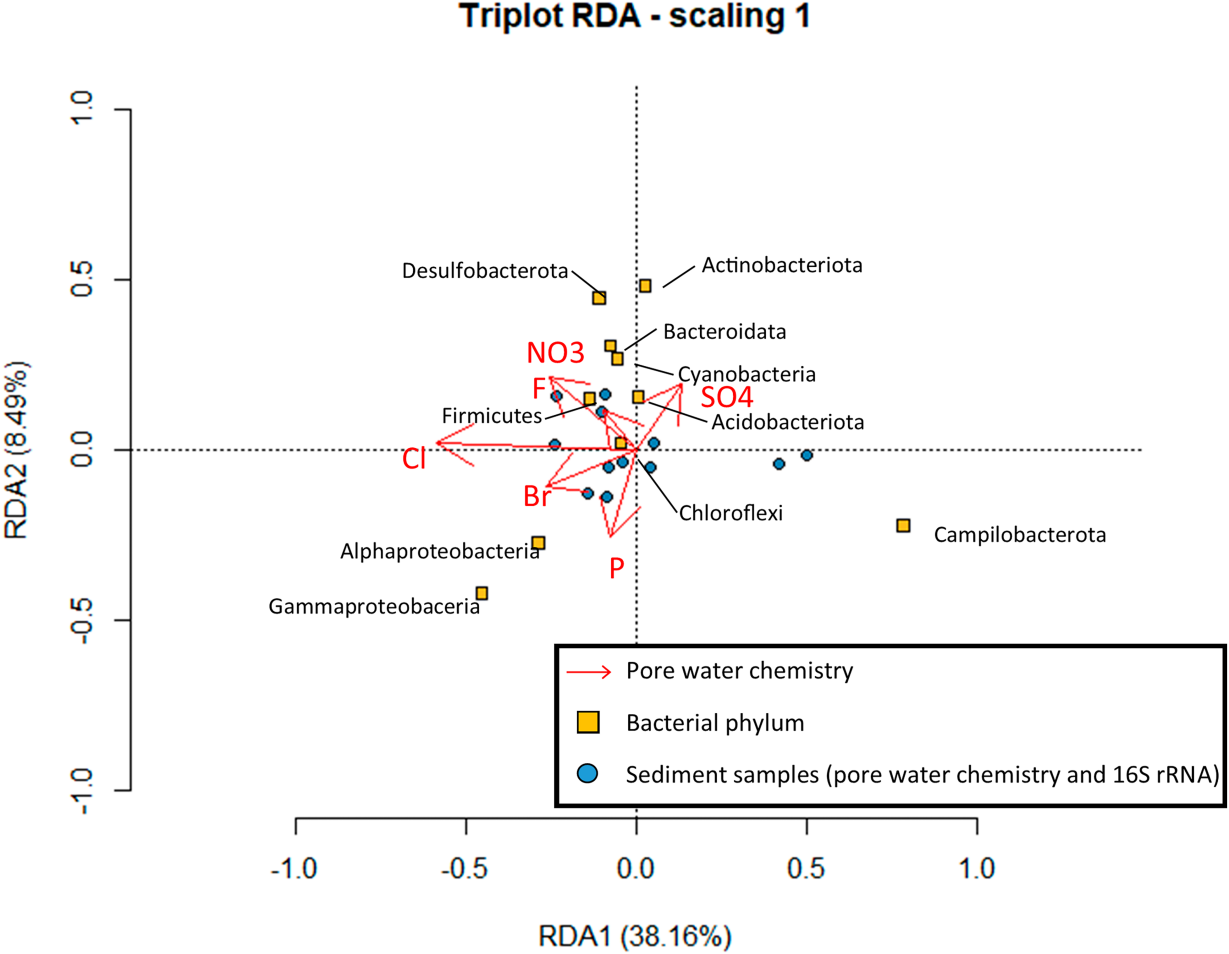
Redundancy analysis (RDA) biplot of environmental parameters and the major phyla identified using 16SrDNA. Br, bromide; Cl, chloride; Fl, fluoride; N, nitrate; P, phosphate; S, sulfate.

Redundancy analysis (Figure 11) identified the correlation between water chemistry and microbial communities in the studied sites. A strong positive correlation of sulfate and nitrate concentration with Campilobacterota in the deeper sediments (during both seasons) in Southend was observed. The microbial community at this site is inferred to have been strongly influenced by storm sewerage derived contamination. Elevated sulfate concentrations in summer pore water samples can be attributed to the admixiture of storm drainage water into the dynamically transported portion of the sediment, as these samples also show elevated nitrate. Another reason for this variation could be linked to the rapid re‐oxidation of bedrock derived sulfide in the surface sediments (Jørgensen et al., 2019). The sulfur‐oxidizing bacteria, *Sulfurovum* sp. and *Sulfurimonas* sp. from Campilobacterota dominated in Southend sediments. These genera have been reported to have the ability to utilize sulfur in their metabolism (Wang et al., 2023). *Sulfurimonas* sp. are known to mediate sulfur oxidation coupled with nitrate reduction.

## DISCUSSION

### Interpretation of corrosion products

The general form of the FTIR spectra shows haematite is ubiquitous in all samples. At Southend, the peaks at 809 and 888 cm^−1^ can be attributed to goethite (α‐FeO(OH)) and that at 1012 cm^−1^ to a minor component of lepidocrocite (γ‐FeO(OH)) (RRUFF database; accessed 10/10/2022), and all arise from Fe–O‐ and Fe–O–H bond stretching modes (Caldeira et al., 2003; Weerasooriya et al., 2010). Related peaks can be resolved in spectra from Shoreham and Newhaven, but the strong development of a peak at 1023 cm^−1^, and lower intensity peak at 1130 cm^−1^ can be attributed to a much higher relative concentration of lepidocrocite, and lower occurrence to below detection levels of goethite. Additional adsorption peaks at 708 cm^−1^ and 861 cm^−1^ can be attributed to Fe–O–O and Fe–O–Fe stretching modes respectively (Caldeira et al., 2003; Rouchon et al., 2012; Weerasooriya et al., 2010). The peaks at 861 and 781 cm^−1^ may be specific to Fe–OH lattice vibrational modes in green rust (Fe^II^_4_Fe^III^_2_(OH_12_)SO_4_·8H_2_O) (Peulon et al., 2003). The peak at 781 cm^−1^ may also be overlapped by a monodentate SO_4_^2−^ peak at 791 cm^−1^ (Usher et al., 2005). Sulfate‐specific adsorption peaks are observed at 874 cm^−1^ (HSO_4_^−^), 992 cm^−1^ (observed in amarantite (Fe_2_(SO_4_)_2_O·7H_2_O) – RRUFF database, accessed 10/10/2022; attributable to the bidentate SO_4_^2−^ symmetrical stretch) (Borda et al., 2004; Rouchon et al., 2012; Usher et al., 2005) and 1095 cm^−1^ (monodentate sulfate, observed in rozenite (FeSO_4_·4H_2_O) – RRUFF database, accessed 10/10/2022; attributable to the monodentate SO_4_^2−^ asymmetric stretch) (Borda et al., 2004; Peulon et al., 2003; Usher et al., 2005). The broad adsorption band from 1400–1500 cm^−1^ arises from carbonate in shell aragonite and calcite (1430 cm^−1^, 1475 cm^−1^; Caldeira et al., 2003) and Fe hydroxide gels (Chernyshova, 2003; Hassan et al., 2018), and the minor peak at 1627 cm^−1^ is from water. Peak identifications are summarized in Table 2. The spectra are also potentially affected by adsorption by polysaccharides from biofilms developed on steel and corrosion surfaces. Measurements of reagent grade sodium alginate indicate broad adsorption peaks in the range 1670–1560 cm^−1^, around 1408 cm^−1^, from 1290–1230 cm^−1^, from 1070–1020 cm^−1^, at 1029 cm^−1^, at 886 cm^−1^ and at 808 cm^−1^. However, where the iron and sulfur compound adsorption peaks identified above coincide with alginate peaks, other corresponding peaks from the alginate spectrum do not occur, and so we are confident in the interpretation in terms of iron oxide and sulfsulfur compounds.

**TABLE 2:** Interpretation of corrosion products using FTIR.

Absorption band (cm^−1^)	Group/mode	Mineral	Source
Absorbance <750 cm^−1^		Haematite/Magnetite	RRUFF database
708	Fe‐O‐O‐ or SO_4_^2−^ asymmetrical bend	Green rust	Caldeira et al. (2003), Rouchon et al. (2012), Weerasooriya et al. (2010)
746		Lepidocrocite	RRUFF database
781	Fe‐O‐Fe/Lattice Fe‐OH	Green rust	Peulon et al. (2003)
791	SO_4_ monodentate		Usher et al. (2005)
809		Goethite	RRUFF database
861	Fe‐O‐Fe/Lattice Fe‐OH	Green rust	Peulon et al. (2003)
874	HSO_4_^2−^	Green rust	Usher et al. (2005)
888		Goethite	RRUFF database
992	SO_4_^2−^ bidentate	Amarantite	Borda et al. (2003), Rouchon et al. (2012), RRUFF database
1012		Lepidocrocite	RRUFF database
1023		Lepidocrocite	RRUFF database
1095	SO_4_ monodentate	Rozenite	Borda et al. (2003), RRUFF database, Usher et al. (2005)
1130		Lepidocrocite	RRUFF database
1390		Fe II hydroxide gel	Chernyshova (2003)
1430	Carbonate (CO_3_^2−^)	Aragonite/calcite	Caldeira et al. (2003)
1444	Fe‐OH bend	Fe III hydroxide gel	Hassan et al. (2018)
1475	Carbonate (CO_3_^2−^)	Aragonite	RRUFF database
1627	Water		Shahabi‐Navid et al. (2020)

Overall, these data are consistent with the presence of sulfate green rusts and a greater component of lepidocrocite in the samples from Shoreham and Newhaven, compared to those from Southend. Association of green rust formation and dissimilatory sulfur reductase enzyme was reported in ALWC by Pineau et al. (2008). Lepidocrocite has also been proposed to be diagnostic of microbially influenced low water corrosion (Pineau et al., 2008) and may form by the oxidation of green rusts (Cornell & Schwertmann, 2003). The presence of carbonate is related to residual material from the macrobiota (barnacles, the crustacean *Semibalanus balanoides*) which colonized the steel surface prior to full development of corrosion blisters. The barnacle layer may be responsible for the formation of anoxic micro‐environments at the steel surface that allowed for colonization by sulfate‐reducing prokaryotes. Sulfide oxidation results in an acid microenvironment that dissolves shell material. The adsorption peaks from sulfur phases are poorly developed or absent in the Southend samples, which are dominated by goethite with significantly less adsorption from lepidocrocite. Goethite typically forms as a corrosion product via dehydration of Fe(OH)_3_ gels and aqueous complexes (Cornell & Schwertmann, 2003). The absence of sulfur compounds at Southend is supported by depth profiling by X‐ray photoelectron spectroscopy (Smith et al., 2024). The results support the association between ALWC and the microbial sulfur cycle via the generation of a highly acid microenvironment.

### Microbial community structure and nutrient composition

Our understanding of MIC and the biogeochemical sulfur cycle occurring in marine environments has advanced greatly in recent years. This can be linked to the introduction of new techniques and approaches based on DNA and RNA analysis. The development of high‐throughput DNA sequencing techniques and the rapidly growing database of DNA sequence information have broadened our knowledge of microbial ecology in various habitats. Metagenetic and metagenomic approaches have led to the discovery of many uncultured microbes from various environments and elucidated their role in biogeochemical cycles (Anantharaman et al., 2018). However, we are still far from consolidating the importance of a high proportion of microbial diversity, and the role of those species in sulfur cycle and MIC in marine engineered structures.

In the present study, samples from three different sites in the UK were analysed to understand the mutual influence between the marine sediment and seawater bacterial populations and MIC (particularly ALWC) in marine structures. The results revealed that even though the bacterial community structure in sediment samples is comparable in all the three sites, at Southend‐on‐Sea the relative abundance of Campilobacterota was higher during both seasons, compared to the other two sites with a higher proportion of Proteobacteria. The relatively high proportion of sulfur‐oxidizing bacteria along with high sulfate and nitrate concentration in pore water from Southend site may relate to the ability of sulfur‐oxidizing bacteria to use nitrate as an electron acceptor during the oxidation process (Chen et al., 2008; Li et al., 2021; Manconi et al., 2006). The presence of Acrobacteraceae, a dominant family identified from Southend, suggests a link to anthropogenic activity as high proportion of Acrobacters in environmental samples and water sources indicates faecal pollution or sewage contamination (Venâncio et al., 2022). These organisms have been identified at oxic–anoxic interfaces, where the organism has been noted to oxidize sulfide to sulfur, perhaps in competition with other sulfur‐metabolizing species (Sievert et al., 2007). The overall higher proportion of sulfur‐oxidizing species at Southend may relate to anthropogenic influence. Water column analyses from Autumn 2021 show a large excess of sulfate over concentrations expected from seawater‐river mixing, which can be related to storm sewage release into the estuarine system. In contrast, pore fluid analyses from Southend show very little evidence for the action of sulfate‐reducing bacteria in bed sediment. *Candidatus electrothrix* capable of long distance electron transport identified from Southend surface sediments during winter season belongs to Desulfobulbaceae, and their winter–spring dominance was already reported. This species is abundant in many seasonal hypoxic basins, which, in marine habitats, may be due to anthropogenic nutrient input (Seitaj et al., 2015). The sulfur‐oxidizing *Sulfurimonas*, one of the most abundant genera, is capable of thiosulfate production in subsurface environments which can induce corrosion on metals (Lahme et al., 2020). In the two intertidal sites, Newhaven and Shoreham, the relative proportion of sulfur‐oxidizing bacteria in sediment was lower. The dominant bacteria genera at these sites are Proteobacteria (mainly Alpha‐ and Gammaproteobacteria). The ubiquity of Proteobacteria as the major taxonomic group associated with MIC has already been reported (Li et al., 2017; López et al., 2006; Phan et al., 2020). Members of this group are known to be pioneer ‘surface colonizers’ and ‘biofilm formers’, which facilitates the establishment of a subsequent more diverse population (Dang et al., 2008; Li et al., 2017; Slightom & Buchan, 2009). Rhodobacterales dominated among the Alphaproteobacteria, with most of the genera being not identified, along with *Sulfitobacter* sp., *Thioclava* sp. and *Yoonia‐Loktanella* sp. Most of the genera identified from Gammaproteobacteria are mesophiles such as *Shewanella* sp., *Alcanivorax* sp., *Halomonas* sp. and *Marinomonas* sp. and have been reported to be associated with marine habitats (Salgar‐Chaparro et al., 2020). Even though *Shewanella* sp. are frequently reported for their Fe(III)‐reducing properties (Philips et al., 2018) and their involvement in MIC (McLeod et al., 1998, 2002), their broad metabolic capability stated by (Miller et al., 2018) suggests their role in MIC through different mechanisms such as EET‐MIC (extracellular electron transfer MIC) and CMIC (chemical MIC). The only genus that was identified from Zetaproteobacteria was *Mariprofundus* sp. Members of this genus have been reported to be Fe‐oxidizing species, as well as potential involvement in iron metabolism, the taxa have often been recognized for the production of iron oxyhydroxides.

A majority of Desulfobacterota are SRBs (Waite et al., 2020), and this was identified as one of the most abundant phyla, present in all the three studied sites. Based on pure culture‐corrosion studies, along with the fact that anoxic sulfur‐rich environments promote corrosion where iron sulfide is the main corrosion product, SRBs are considered to be the main causative organism of MIC (Enning & Garrelfs, 2014). The mode of action of SRBs can be either by direct withdrawal of electrons from metal surfaces (‘electrical microbially influenced corrosion’; EMIC) or by the chemical action of hydrogen sulfide produced because of dissimilatory sulfate reduction (‘chemical microbially influenced corrosion’; CMIC). BSA or biogenic sulfuric acid corrosion caused by the combined action of SRBs and SOBs has been extensively studied for concrete corrosion and in sewerage and wastewater treatment facilities, but not reported widely for marine steel structure corrosion (Chaudhari et al., 2022; Huber et al., 2016; Yang et al., 2018). We previously identified this type of corrosion in ALWC on steel in estuarine and marine structures and reported in (Smith et al., 2019). The involvement of SRBs and SOBs in corrosion at Shoreham and Newhaven is attested to by both the 16sRNA data presented here and the chemistry of corrosion products. Sulfuric acid can be produced by the oxidation of pyrite, in the presence of water and oxygen (Fellowes & Hagan, 2003) and is a critical factor in the accelerated corrosion rates of this form of MIC. The comparison of 16sRNA data with mineralogical data on corrosion products and environmental chemical data indicates that despite their presence in the local environment, sulfate‐reducing bacteria are not active in bed sediment or corrosion at SE because of the more oxygenated environment and dynamic sediment transport, and the corrosion is dominated by abiotic mechanisms or direct EMIC. Our research indicates that the mechanism of action of microbes in accelerated intertidal corrosion types is almost certainly due to a combination of CMIC and BSA (the corrosion process initiates due to the metabolic products of microbes). However, given the microbial population diversity, the direct participation of microbes (EMIC) cannot be ruled out, along with the involvement of many different populations from other bacterial taxa. This results in the formation of different corrosion by‐products on the surface of metals. The rapid corrosion rates seen in ALWC are a result of the highly acid environment produced by the combined action of SRBs and SOPs in corrosion blisters. The accelerated corrosion rate is a function of complex microbial communities and cannot be attributed to a single organism. The results here are comparable in some respects to those of Phan et al. (2020) from orange tubercles from Australia, where Proteobacteria were shown to dominate corrosion communities. They emphasized the importance of Deltaproteobacteria in the development of ALWC, which have been reclassified into 3 phyla (Langwig et al., 2022), including Desulfobacterota which have been part of the focus of this study. The relative abundance of phyla, and the precise species present vary, however, indicating that the overall mechanism of microbial involvement in ALWC is related to the sulfur cycle metabolic pathways, with a high degree of functional redundancy between causative organisms, and not to specific causative organisms.

Many studies have reported the influence of temperature on microbial biodiversity (García et al., 2018; Koizumi & Nara, 2020; Luo et al., 2019). In this study, there was a significant difference in bacterial community structure in sea water during the two seasons, at all the three sites. These findings suggest that temperature and precipitation are key abiotic factors determining microbial community development in the environment, and that anthropogenic activities can influence the microbial diversity, thus potentially affecting corrosion. The microbial population within the corrosion blisters from all the three sites were dominated by Proteobacteria. The finding that the microbial diversity of corrosion blisters is similar to the communities in anoxic bed sediments is in agreement with the hypothesis that microorganisms in marine habitats reach the metal surface to initiate corrosion through sediments suspended in water. Close comparison between sediment and corrosion populations (Figure 4B) suggests associated bacterial populations are derived from local bed sediment, mixed to varying degrees with those with the water column. This is in contrast to the conclusion of Phan et al. (2019) that there was no strong evidence of local sediment as the source of corrosion tubercle microbes.

The identification method developed to identify SRBs using qPCR‐HRM is rapid and cost effective, which eliminates the need of sequencing to determine the variants. The variants can be identified visually by the shift in temperature of the melting curve. Molecular‐based approaches based on quantitative measurements of *dsrAB* genes are being widely used for the presence of SRBs in environmental samples. About 13 lineages of this gene have been identified at bacterial family level by (Müller et al., 2015; Pester et al., 2012). The bacterial genomes possessing the gene at higher proportions can be affiliated to Desulfobacterota> Pseudomonadota (Alphaproteobacteria, Gammaproteobacteria) > Bacteroidota > Acidobacteriota (Diao et al., 2023). It is noteworthy that these phyla represent the highest proportion of abundances in our analysis. It is also reported that more than 99% of SRP diversity is represented by uncultured microorganisms without taxonomic assignment (Diao et al., 2023). In our HRM‐qPCR method, we identified some *dsr*B variants only in Southend samples. As reported by (Diao et al., 2023), these can be attributed to thiosulfate‐reducing bacteria that possess the *dsr* pathway (Campylobacterota), as this phylum dominates in this particular study location. Further research is needed to fully understand the diversity of *dsr* genes observed in our developed technique. The developed method is promising as a rapid, low‐cost technique for identifying SRBs in environmental samples.

Though sulfur‐reducing bacteria and iron‐oxidizing bacteria are the focus of much attention in discussions about MIC, our findings about the microbial diversity in corrosion blisters show that members from Proteobacteria, Campilobacterota, Actinobacteriota, etc. also contribute to the process. These findings expand our current understanding on bacterial population structure and function in MIC, respecting the hypoxic and nonhypoxic areas of the studied sites in the UK. Meanwhile, a huge proportion of bacteria found in corrosion samples and marine environments are still unclassified and not studied. It is evident that the extent of MIC depends on a variety of environmental factors with the participation of a diverse group of microorganisms. Efforts to study the direct relation between microbes and MIC employing a single bacterial taxon under laboratory conditions, also require community level investigations in order to fully understand MIC mechanisms in natural and engineered environments. An alternative to identify corrosion mechanisms at the community level is to enumerate the microbial population through metagenetic or amplicon analysis and metagenomic studies.

## CONCLUSIONS

Recent advances in the field of DNA and RNA analysis techniques have greatly contributed to our comprehension of microbial communities, and their role in the biogeochemical sulfur cycle within marine environments. Despite these remarkable achievements, a substantial portion of microbial diversity remains unexplored, particularly in relation to their significance in marine engineered structures and their contribution to MIC in these specific settings. The investigation carried out across three sites in the United Kingdom has revealed distinct bacterial communities in marine sediment. The conducted study encompassed a comparative analysis of 16SrRNA data, mineralogical data on corrosion products, and environmental chemical data. The research concluded that corrosion mechanisms could originate from either CMIC or BSA, accompanied by the active involvement of various bacterial taxa. The generation of sulfuric acid by the action of both sulfate‐reducing and sulfide‐oxidizing taxa is critical in the generation of accelerated corrosion rates. Consequently, diverse corrosion by‐products is formed in intertidal environments, depending on the activity of sulfur cycling bacteria in corrosion. The accelerated corrosion rates observed in the intertidal zone (ALWC) are a function of complex microbial communities, rather than a single causative organism. The detection of lepidocrocite and sulfate green rust through FTIR analysis of corrosion blisters may be diagnostic of microbially influenced corrosion (MIC). The strong correlation in microbial diversity between corrosion blisters and local, subsurface, sediments in the two sites with identified ALWC indicates that the local sediment is the source for the corrosion communities. The method of rapid identification that has been developed for the purpose of identifying SRBs using qPCR‐HRM has shown great promise in efficiently identifying SRBs in environmental samples. The qPCR‐HRM results confirm the presence of the same *dsrB* gene variants in sediment and corrosion at the two sites with confirmed ALWC, but with distinct variants at the ALWC free site.

## AUTHOR CONTRIBUTIONS

**Biji Shibulal:** Writing – original draft; methodology; data curation; investigation; formal analysis; conceptualization. **Martin Peter Smith:** Writing – review and editing; conceptualization; funding acquisition; investigation; supervision; formal analysis. **Ian Cooper:** Writing – review and editing; methodology; investigation; conceptualization. **Heidi Marie Burgess:** Funding acquisition; conceptualization; investigation. **Norman Moles:** Methodology; writing – review and editing; investigation. **Alison Willows:** Writing – review and editing; investigation.

## CONFLICT OF INTEREST STATEMENT

The authors declare no conflict of interests.

## CODE AVAILABILITY

The R codes for amplicon data analysis are publically available at https://github.com/Bijishibulal/SOCORRO_Amplicon_Analysis.

## Data Availability

Amplicon sequencing raw data that support the findings of this study have been deposited in GenBank and can be accessed from NCBI SRA (Bioproject ID: PRJNA974398); https://www.ncbi.nlm.nih.gov/sra/PRJNA974398. The supplemental files can be accessed using the link: https://doi.org/10.6084/m9.figshare.26527144.v1.
